# Glomus tumor presenting as complex regional pain syndrome of the left upper limb: a case report

**DOI:** 10.1186/s13256-015-0793-3

**Published:** 2015-12-29

**Authors:** Chege Macharia, Peter M. Nthumba

**Affiliations:** 1 General Surgery, AIC Kijabe Hospital, Kijabe, Kenya, Africa; 2 Plastic, Reconstructive and Hand Surgery Unit, AIC Kijabe Hospital, Kijabe, 00220 Kenya, Africa; 3 University of Bern, Bern, Switzerland; 4 London School of Hygiene & Tropical Medicine, London, UK

**Keywords:** Complex regional pain syndrome, Glomus tumor, Rare, Sub-Saharan Africa

## Abstract

**Background:**

Glomus tumors of the hand are rare, benign but debilitating neoplasms arising from the neuromyoarterial glomus body. They may present a diagnostic dilemma, and take years with multiple consultations and investigations before an appropriate diagnosis is made, but once a diagnosis is made, surgical excision is curative.

**Case presentation:**

This is a case presentation of a 35-year-old African man who presented with complex regional pain syndrome of his left upper extremity, whose genesis was found to be a glomus tumor of the pulp of his left middle finger. Surgical excision resulted in resolution of the chronic regional pain syndrome and a return to a normal lifestyle. Chronic regional pain syndrome is a rare presentation of a glomus tumor, which has only been previously reported in patients with neurofibromatosis type 1, and one patient who did not have neurofibromatosis.

**Conclusions:**

Patients with glomus tumors may spend many years in pain and distress because of misdiagnosis. Sensitization and education of both the public and health care workers will help in early diagnosis and treatment of this otherwise potentially disabling pathology for which surgical excision is curative.

## Background

Glomus tumors are rare benign hamartomas that account for 1 to 5 % of soft tumors of the hand. They arise from normal glomus apparatus located in subcutaneous tissue. The glomus body is a contractile neuromyoarterial receptor that controls blood pressure and temperature by regulating peripheral blood flow. Normal glomus bodies are distributed throughout the body in the reticular dermis, but are concentrated in the tips of digits, especially subungually [1]. A glomus tumor results from hyperplasia of any part of the glomus apparatus, and can therefore occur anywhere in the body.

The classic symptom presentation is a triad of pinpoint tenderness, paroxysmal pain and hypersensitivity to cold [1]. Changes in temperature cause contraction of myofilaments resulting in an increase in intracapsular pressure and cause the perception of pain [1]. The rarity of glomus tumors makes them difficult to diagnose, and patients may spend years seeing different doctors before the right diagnosis is made, and appropriate treatment offered.

We present the case report of a 35-year-old African man who presented with an 8-year history of severe left upper extremity pain and paresthesia and exquisite middle finger pain; these symptoms presented a diagnostic dilemma. Surgical exploration of his finger revealed a glomus tumor, and surgical excision led to complete resolution of all his symptoms.

## Case presentation

A 35-year-old African man presented with an 8-year history of left upper limb pain. The pain was so severe that he was unable to sleep at night. He complained of almost incessant pain and paresthesia from the left side of his neck, and along his entire left upper limb. Touching him anywhere on his limb and neck would elicit a burning pain that radiated along his entire upper extremity. There was no antecedent history of trauma, and he did not have any features of neurofibromatosis type 1.

On further probing, he reported that the pain had initially started from the tip of his left middle fingertip. Over time, the pain had grown worse, and extended to involve the entire limb.

The pain was aggravated by cold weather, lifting of heavy weights with his left hand and direct pressure or trauma to his left middle finger. It was relieved by rubber band tourniquet (self-applied) and by rubbing either side of his finger on a rough cloth or other rough surface. During the 8-year period, he was managed for multiple different diagnoses including neurogenic pain, neuroma, gout and thoracic outlet syndrome. He underwent multiple diagnostic tests including computed tomography (CT) scan and magnetic resonance imaging (MRI) imaging of his spine and brachial plexus. He had a total of 12 stellate ganglion blocks administered over the 8 years without a resolution of symptoms.

On examination, he had neck tenderness along the brachial plexus roots, and along his entire left upper limb. His left middle finger was visibly thickened and dry, with exquisite tenderness over the entire pulp, precluding further examination.

A diagnosis of chronic regional pain syndrome (CRPS), most likely secondary to a glomus tumor, was made. Previous MRI images were inconclusive, and we did not see the need to get new ones. We proposed an exploration of his left middle fingertip, and informed him that the finding and excision of a glomus tumor would be curative.

**Fig. 1:**
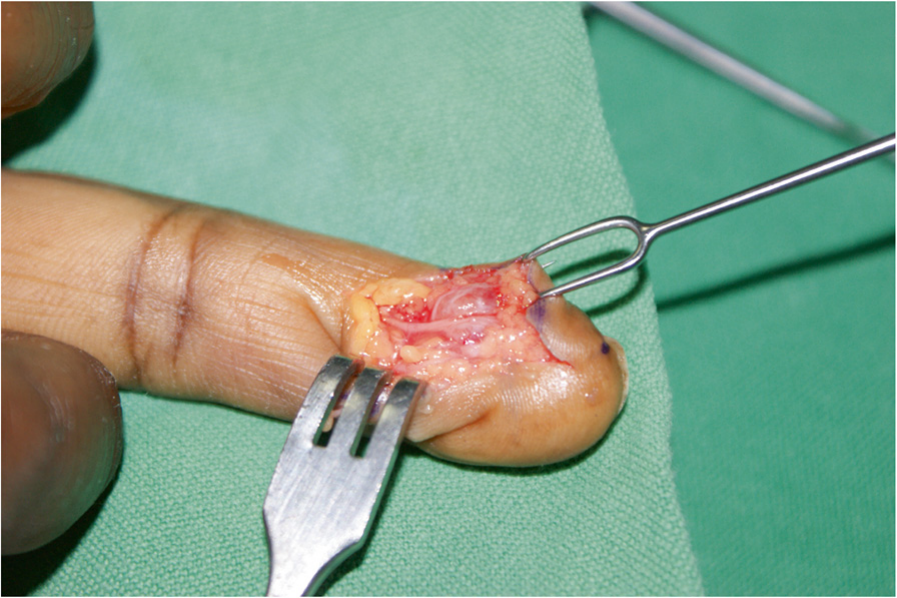
Glomus tumor in the fingertip. Note intimate relationship with digital nerve

He consented to surgery, which was performed under a regional block and tourniquet, under loupe magnification. A 10×6 mm tumor was found adjacent to bone and excised (Fig. 1).

**Fig. 2:**
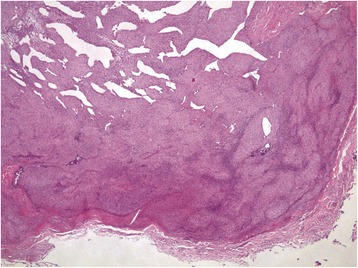
The tumor is sharply circumscribed by a fibrous pseudocapsule. Centrally ectatic, branching vessels are surrounded by glomus cells (hematoxylin and eosin stain, 40×)

**Fig. 3:**
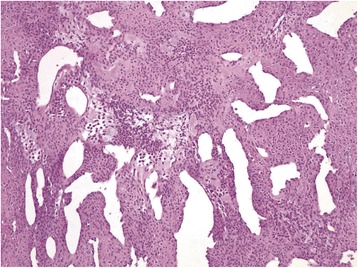
Irregular, branching ectatic vessels surrounded by glomus cells in variable amounts of fibromyxoid stroma. (Hematoxylin and eosin stain, 100×)

**Fig. 4:**
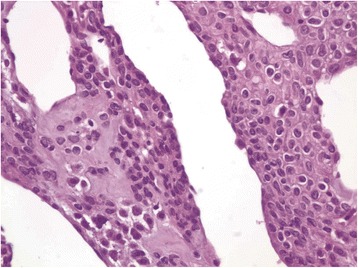
Glomus cells in fibromyxoid stroma around vessels. Nuclei are uniform, round, “punched out,” without atypia or mitotic activity. (Hematoxylin and eosin stain, 400×)

**Fig. 5:**
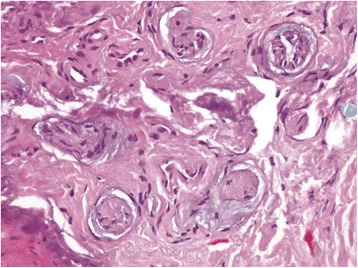
Within fibrous pseudocapsule are numerous small nerve twigs, correlating with the clinical symptom of intense pain. (Hematoxylin and eosin stain, 400×)

His postoperative recovery was uneventful; he reported uninterrupted sleep for the first time in 8 years. So happy was he with his outcome, and so concerned for others that might be in pain like he had been, that he approached a local newspaper where he narrated his experiences [2]. The histopathology was reported as a glomus tumor (Figs. 2, 3, 4 and 5).

## Discussion

Glomus tumors are rare, benign vascular tumors that occur anywhere in the body. Of these, 75 % occur in the hand. Fifty percent to 90 % of glomus tumors in the hand are subungal. These tumors usually present in people between the ages of 30 and 50 years, and have a male to female ratio of 2:1.

Glomus tumors may be solitary or multiple; solitary lesions are encapsulated and most commonly subungal, while multiple tumors are unencapsulated and rarely subungal. Multiple tumors present in 2 to 3 % of cases, are usually asymptomatic and present earlier in life [1].

The diagnosis is primarily clinical; glomus tumors classically present with a triad of severe intermittent pain, localized tenderness and sensitivity to cold (Table 1) [3]. The tumors are usually less than 1 cm in their greatest dimension at the time of presentation. The patient will normally not permit palpation of the tumor because of the tenderness. In addition, subungal glomus tumors may present with an area of blue discoloration under the nail with or without nail deformity.

**Table 1:** Glomus tumor: clinical tests, symptoms and sensitivities. Adapted from [3]

Test		Symptom	Sensitivity	Specificity	Accuracy
Love’s pin test	Pinhead pressure over tumor	Severe pain	100 %	0 %	78 %
Hildreth’s test	Tourniquet applied and Love’s test	No pain	71.4 %	100 %	78 %
Cold sensitivity test	Cold water over hand	Severe pain	100 %	100 %	100 %

Delay in the diagnosis in the patient presented here led to an aggravation of the symptoms and the development of CRPS over a period of 8 years. A diagnosis of CRPS may be made if the following conditions are met after the exclusion of other diagnoses: a patient presenting with spontaneous pain or hyperalgesia/hyperesthesia not limited to a single nerve territory and disproportionate to the inciting event; and edema, skin blood flow (temperature) or sudomotor abnormalities, motor symptoms or trophic changes on the affected limb. In our patient, the initial insult was an undiagnosed glomus tumor that was repeatedly stimulated; he maladjusted for the resultant pain, causing him to experience excessive pain and hyperesthesia involving his entire upper limb. His pain was so severe and so extensively distributed over his upper extremity, that he had several MRIs and stellate ganglion blocks in an attempt to control the pain. He had trophic changes over his fingertips, most pronounced on his finger with the glomus tumor.

Thus, for most of the 8 years, he was managed for CRPS that progressively worsened. At the time of his initial visit to our institution, deciphering the initial pathology was nearly impossible. However, a careful review of the chronology of the illness pointed to the likelihood of a glomus tumor. Surgical exploration was performed based on clinical suspicion, as accompanying MRIs did not reveal any pathology in his fingertip.

We found one case report of CRPS in a patient with a glomus tumor without neurofibromatosis type 1 in the English literature; all other reports were of patients with neurofibromatosis type 1 [4, 5]. Patients with CRPS may have a glomus tumor as the cause of their illness, and it needs to be added to the differential diagnoses, a consideration that would shorten the lag time between presentation and treatment.

The diagnosis of a glomus tumor may be aided by the use of several imaging modalities including plain radiography, Doppler ultrasonography and MRI. Plain radiographs may detect some concavity in the bone in patients with tumors that are adjacent to bone and present for a significant duration of time. Vandenberghe and De Smet described a technique in which lateral finger radiographs of two opposing fingertips pointing towards each other are taken, allowing for a comparative view of both fingertips; thus, even subtle cortical scalloping of the affected side can be detected. This sign, however, is present in only 22 % of patients [6].

CT scan imaging is not a useful modality for diagnosis. In expert hands, ultrasonography especially with color Doppler may identify these lesions.

High-resolution MRI is a sensitive but not specific diagnostic tool; it is capable of detecting lesions as small as 1 mm. MRI is therefore a useful radiological adjunct to clinical examination, especially in those cases with nonspecific symptoms. However, an MRI does not always show a glomus tumor, and a strong clinical suspicion should therefore lead to surgical exploration even in the face of a negative MRI, as was the case in our patient [1].

Histological examination of submitted tissue provides the definitive diagnosis. Glomus tumors are made of three primary cell/tissue types: glomus cells, vasculature and smooth muscle cells. There are three histological subtypes, which are based on the predominant cellular pattern within each tumor. Solid glomus tumors have poor vasculature and a scant smooth muscle component, whereas glomangioma tumors have a prominent vascular component. Glomangiomyoma tumors have prominent vascular and smooth muscle components. Solid glomus tumor is the most common variant (75 %) followed by glomangioma (20 %) and glomangiomyoma (5 %) [7]. Our patient had a glomangioma variant.

Treatment of glomus tumors is by surgical excision. The tumor is ideally excised along with its capsule (Fig. 1); failure to remove the whole tumor will lead to a recurrence. A complete excision results in immediate and complete cure, and in the present case report, surgery allowed the patient to return to a normal lifestyle, including sleep.

Recurrence rates of between 1 % and 8 % have been reported after surgical excision. Recurrence within weeks to months of surgery is thought to be due to inadequate excision, while recurrence occurring more than 2 years after surgery is regarded as the result of new tumors [1].

## Conclusions

Patients with glomus tumors may spend many years in pain and distress because of misdiagnosis. Unnecessary and costly investigations add to the delay in the correct diagnosis and appropriate treatment. Sensitization and education of both the public and health care workers will help in early diagnosis and treatment of this otherwise potentially disabling pathology, which can be cured by surgical excision. The treatment of CRPS is complex and difficult. Although rare, this report affirms that clinicians can add glomus tumor to the differential diagnoses of causes of CRPS.

## Consent

Written informed consent was obtained from the patient for the publication of this case report and any accompanying images. A copy of the written consent is available for review by the Editor-in-Chief of this journal. The care and reporting conform fully to the Helsinki Declaration.

## Abbreviations

CRPS: Chronic regional pain syndrome
CT: Computed tomography
MRI: Magnetic resonance imaging
